# Preliminary Study on the Development of a Transmission Model for Canine Distemper Virus in Wildlife Populations Using Heat Mapping and the Basic Reproduction Number

**DOI:** 10.3390/vetsci13010083

**Published:** 2026-01-14

**Authors:** Bryan Andrew Lazarus, Muhammad Farris Mohd Sadali, Farina Mustaffa Kamal, Khor Kuan Hua, Ridhwan Abdul Wahab, Mohd Arifin Kaderi, Mohd Lutfi Abdullah, Tengku Rinalfi Putra Tengku Azizan, Hafandi Ahmad

**Affiliations:** 1 Department of Veterinary Preclinical Science, Faculty of Veterinary Medicine, University Putra Malaysia (UPM), Serdang 43400, Malaysia; bryanlazarus94@gmail.com (B.A.L.);; 2 Department of Veterinary Pathology and Microbiology, University Putra Malaysia (UPM), Serdang 43400, Malaysia; farrissadali98@gmail.com (M.F.M.S.); farina@upm.edu.my (F.M.K.); 3 Department of Clinical Studies, Faculty of Veterinary Medicine, University Putra Malaysia (UPM), Serdang 43400, Malaysia; khkhor@upm.edu.my; 4 Fakulti of Medicine, MAHSA University, Selangor 42610, Malaysia; ridhwan@mahsa.edu.my; 5 Department of Biomedical Science, Kulliyyah of Allied Health Sciences, Kuantan Campus, International Islamic University Malaysia, Jalan Sultan Ahmad Shah, Kuantan 25200, Malaysia; 6 National Wildlife Forensic Laboratory, Ex-Situ Conservation Division, Department of Wildlife and National Parks Peninsular Malaysia, Kuala Lumpur 56100, Malaysia

**Keywords:** CDV, wildlife, transmission, basic reproductive number, conservation

## Abstract

Canine Distemper Virus (CDV) is a highly contagious disease that affects a wide range of wildlife species, posing a serious threat to biodiversity and conservation efforts. Despite its ecological significance, the transmission dynamics of CDV in wildlife remain poorly understood, especially in tropical ecosystems. One of the main challenges in studying CDV transmission is the lack of reliable epidemiological data and the difficulty in capturing and monitoring wild animals for surveillance purposes. Thus, this study aims to develop a model to estimate the potential transmission of CDV in wildlife populations using spatial heat mapping and the basic reproduction number (R_0_) as key indicators. A combination of field observation records, environmental data, and reported CDV cases were used to generate predictive heat maps and simulate disease spread across susceptible wildlife hosts. Results showed that certain environmental factors and animal density hotspots significantly contribute to higher transmission potential of CDV. Preliminary results suggest that high-risk zones can be identified based on overlapping wildlife movement corridors and human interface areas. This modeling approach offers a valuable tool to guide targeted monitoring, early detection and conservation strategies against CDV outbreaks in wildlife.

## Simple Summary

Canine Distemper Virus (CDV) is a contagious disease that affects many wildlife species and threatens biodiversity. However, viral spread in wildlife, especially in tropical areas, is not well understood due to limited data and difficulties in monitoring wild animals. This study developed a model to estimate the CDV transmission using spatial heat maps and basic reproduction number (R_0_) in which field observations, environmental data and reported CDV cases were used to predict areas with higher transmission risk. The results showed that environmental factors, animal density and areas where wildlife and humans interact increase the risk of CDV transmission. This model is useful as a preliminary tool to identify high-risk zones and support targeted monitoring, early detection and evidence-based conservation efforts.

## 1. Introduction

Canine distemper Virus (CDV) is a highly contagious viral disease that affects a wide range of carnivores. This multi-host virus has been a causative agent for the population decline of species such as African wild dogs (*Lycaon pictus*), Santa Catalina Island foxes (*Urocyon littoralis catalinae*) and black footed ferrets (*Mustela nigripes*) [1,2]. Transmission of CDV occurs primarily via the respiratory tract; however, the CDV is also shed in the urine and feces [3]. Whilst endangered species are vulnerable to the effects of CDV, the virus cannot persist in small populations [1]. However, the multi-host–pathogen which is CDV can remain a persistent threat in habitats where the abundance of susceptible small mammal hosts may maintain the virus in an enzootic state acting as reservoir hosts [1]. Species within the reservoir are then able to transmit the virus to endangered hosts as they exhibit normal foraging behavior [4].

As the route of transmission of CDV to wild mammals has not been fully determined, the role of the domestic dog and wild carnivores in the maintenance and transmission of the CDV remains unclear [5]. However, CDV has been shown to maintain in wildlife populations with low levels on infection, suggesting that CDV infections persist in wildlife species within complex reservoir systems [6]. For instance, in the Greater Yellowstone Ecosystem, it has been suggested that the presence of more than one competent host for CDV can greatly increase long-term virus persistence provided the spatial area is smaller [7]. In addition, an agent-based stochastic model revealed that CDV could not be independently sustained in a single population [8]. This suggests that multi-hosts are required for the maintenance of CDV in wildlife species.

It has been reported that CDV was detected in tree shrews *Tupaia glis* of the order Scandentia in Kampung Besul Lama, a novel detection, suggesting that small mammals may play a role as reservoir hosts, maintaining and transmitting the virus [9]. This novel discovery further solidifies the hypothesis that mammals aside from rare species may play a role in the maintenance and transmission of the CDV infection. For instance, in Kampung Besul Lama, CDV was the causative agent for the death of a wild Malayan tiger *Panthera tigris jacksoni* [10], confirming the transmission of CDV to rare species. There are little to no studies of CDV transmission towards wildlife in Malaysia. Thus, the current study utilizes the daily movement range of different captured species and their locations to produce a heatmap illustrating the spatial distribution of small mammal species using the QGIS 3.4.0 Bratislava software in Kampung Besul Lama. This is to visualize the movement of these small species in areas of human settlements and their potential movement and virus transmission into tiger areas. In addition, the heatmap also displays overlap of spatial distribution between taxa. Additionally, the study develops a simple model for the calculation of the basic reproductive number within Kampung Besul Lama, to serve as a preliminary baseline for the sporadic CDV events in the area.

## 2. Materials and Method

### 2.1. Ethical Approval

The permit for wildlife research at Kampung Besul Lama was approved by the Department of Wildlife National Parks (PERHILITAN), Peninsular Malaysia [Reference:100-34/1.24 Jld29(11)]. All animal handlings were conducted according to the principle of Laboratory Animal Care and Use Committee [Institutional Animal Care and Use Committee (IACUC)] approved by Universiti Putra Malaysia (UPM/IACUC/AUP-R092/2017).

### 2.2. Study Period and Location

**Figure 1:**
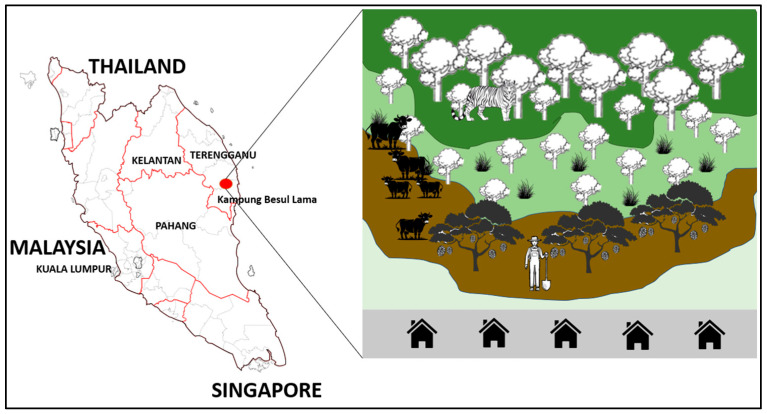
The area of Kampung Besul Lama, consisting of village orchards bordering tropical rainforests, provides a unique environment where there is overlap in the domestic animal and wildlife interface (red oval).

This study was conducted from 19 July 2022 to 10 May 2025 in Kampung Besul Lama, Terengganu. This area was chosen as the location of study due to the first confirmed detection of CDV in a Malayan tiger [9,10]. The area of Kampung Besul Lama (Figure 1), consisting of village orchards bordering tropical rainforests, provides a unique environment where there is overlap in the domestic animal and wildlife interface. The illustration of Kampung Besul Lama uses contrasting colours to represent different land uses and ecological zones. Dark and light green areas represents forested and peri-forested habitats supporting wildlife, whereas the brown sections represent agricultural or disturbed land where human activities occur. The gray strip at the bottom indicates human settlements, highlighting the close proximity and interaction between people, domestic animals and surrounding wildlife.

### 2.3. Wildlife Trapping Using Baited Traps

**Figure 2:**
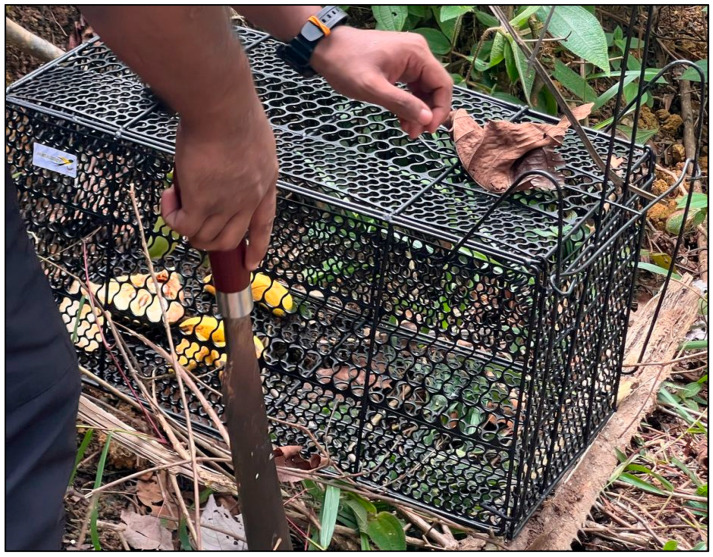
Wildlife trap baited with jackfruit, bananas and oil palm kernel.

**Figure 3:**
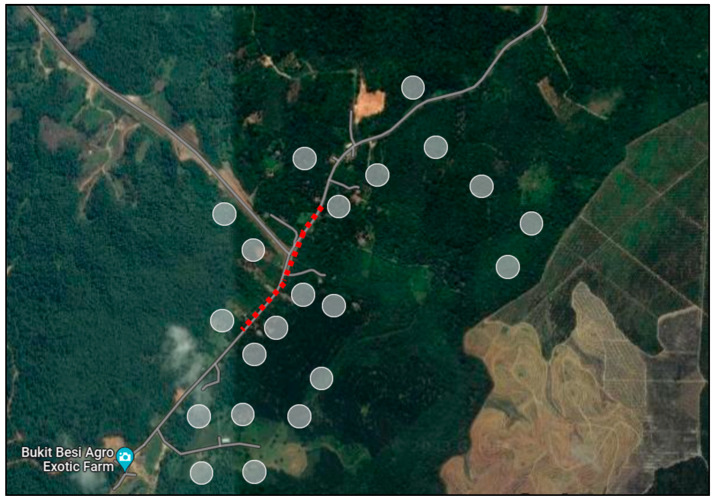
Location of wildlife traps (gray circles) along areas of movement of *Awang Besul* (red line). Image adapted from Google Earth version 10.96.0.1 Multi-threaded.

Spring-live cage traps made of steel with locking mechanisms were used for the small mammal trapping. The spring mechanisms were baited with jackfruit, bananas and oil palm kernels (Figure 2), which shut once triggered; the lock mechanism prevented the animal from escaping. Two sets of wildlife traps were used: (n = 10; 18 cm × 29 cm × 13 cm) was aimed at smaller mammals such as squirrels and rats and (n = 10; 40 cm × 30 cm × 65 cm) was used for larger mammals such as civets, wild cats and other potential mammals. The wildlife traps were placed in peri-forested areas where *Awang Besul*, the first documented Malayan tiger to succumb to CDV, was suspected to have traversed, and orchards (Figure 3). Wildlife traps were also placed along bushes, wildlife paths and areas surrounding chicken coops. The wildlife traps were checked twice daily (morning and evening) and the baits were replaced if damaged or spoiled.

#### 2.3.1. Heatmap Generation

Animal species trapped were identified according to the field guide for the mammals of Southeast Asia [11]. Every successful trapping was recorded and the capture location was georeferenced using a GPS device. Google satellite images were loaded into the software using http://www.google.cn/maps/vt?lyrs=s@189&gl=cn&x={x}&y={y}&z={z} accessed on 22 April 2025. Capture coordinates were systemically recorded according to order and compiled using Google Sheets and loaded into the QGIS 3.4.0 Bratislava software as comma separated values (.csv) files. Then, daily movement and foraging range of each species based on the literature were applied in the QGIS software to generate a heatmap for the visualization of the spatial distribution of the small mammal species. Generation of spatial distribution was performed utilizing estimated foraging range of civets, *Paradoxurus hermaphroditus* and Viverra *tangalunga* at 591.8 m [12], tree shrews *Tupaia glis* at 135 m [13], plantain squirrel *Callosciurus notatus* at 85.1 m [14], gray-bellied squirrel *Callosciurus caniceps* at 71.36 m [15] and rats Rattus rattus at 196.44 m [16]. The heatmap represents the estimation of species movement into forested areas based on their daily movement and capture coordinates. The heatmap of higher intensity (whiter color) depicts higher extensive activities whereas areas of lower color intensity show lower levels of species activity.

#### 2.3.2. Basic Reproductive Number

The basic reproductive number (R_0_ has been described as utilizing the probability of infecting a susceptible individual during one contact, number of new susceptible individuals contacted and the duration of the infections period [17,18,19], and thus, as using the transmission rate (β), number of susceptible individuals (S) and infectious period (D). The infectious period is reciprocal to the recovery rate which means γ = 1/D. R_0_ is usually expressed using recovery rate (γ) because recovery rate is a rate-based measure which aligns with epidemiological models [19].

R_0_ = β × S × D

where

β = transmission rate

S = number of susceptible individuals in population at the start of the epidemic [20]

D = infectious period which will be written as recovery rate γ = 1/D

Thus,

R_o_ = βS/γ

Additionally, the basic reproductive number R_o_ is associated with population density, where greater population density may potentially facilitate higher interactions between individuals, thus sustaining continuation of disease [21]. Thus, the formula introduces the spatial factor A = area, where contact rate is assumed to depend on population density. Thus,

R_o_ = βS/Aγ

where

β = transmission rate

S = number of susceptible individuals in population

A = Area

γ = recovery rate

## 3. Results and Discussion

### 3.1. Species Trapped

The current study shows the species trapped were obtained and georeferenced from the sampling period of 19 July 2022 to 3 December 2025 (Table 1). Wildlife species such as civet species (Asian palm civet *Paradoxurus hermaphroditus* and Malayan civet *Viverra tangalunga*) (n = 14), tree shrews *Tupaia glis* (n = 20), squirrel species (plantain squirrel *Callosciurus notatus*, slender squirrel *Sundasciurus tenuis*, gray-bellied squirrel *Callosciurus caniceps*) (n = 48) and rats (*Rattus rattus* and *Leopoldamys sabanus*) (n = 34) were trapped in Kampung Besul Lama. A previous study reported that species of Viverrids, Rodents and Scandentia such as tree shrews, squirrels and civets are notorious species commonly found in areas of orchards [22]. It has been established that Viverrids such as civets and Rodents such as the squirrel species [23] as well as Scandentia such as tree shrews [9] are susceptible to the Canine Distemper Virus. These species were classified into their respective orders and the study conducted hotspot analysis of the spatial distribution of wildlife species in Kampung Besul Lama while integrating estimated daily movement range into the heatmap function of the QGIS software. Thus, this allowed visualization of the areas of activity and estimation of species movement into adjacent areas.

### 3.2. Heatmap

**Figure 4:**
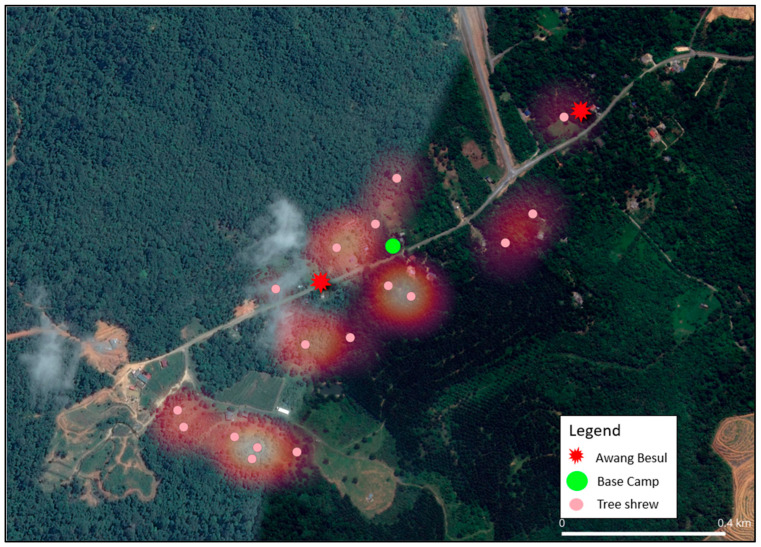
Heatmap representing the distribution and activity density of tree shrews *Tupaia glis* (Scandentia) across the study area. Pink points denote live-capture coordinates, while heatmap gradient reflects relative abundance. The base camp is denoted by the green circle whereas the red starburst represents sightings of *Awang Besul*.

**Figure 5:**
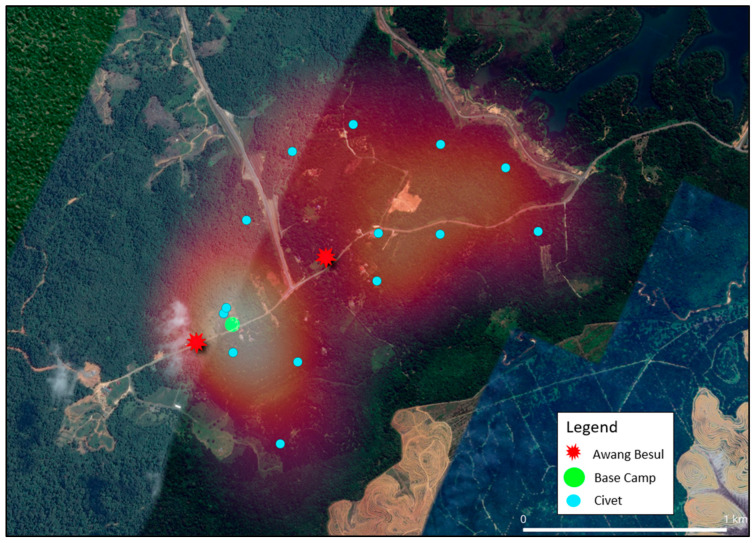
Heatmap illustrating the spatial distribution of civets (Viverridae) activity in Kampung Besul Lama. Blue points indicate live-capture coordinates whereas gradient shading denotes area of activity intensity. The base camp is denoted by the green circle whereas the red starburst represents sightings of *Awang Besul*.

**Figure 6:**
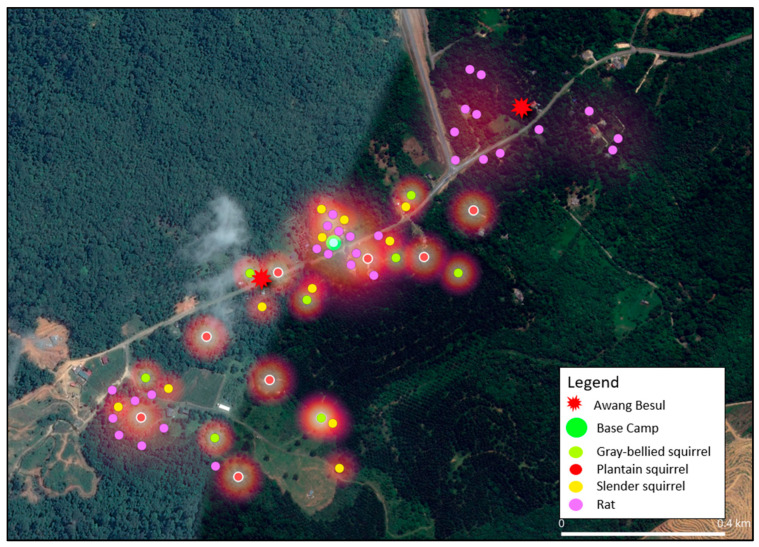
Combined heatmap showing the live-capture locations and activity of Rodents, plantain squirrel *Callosciurus notatus*, slender squirrel *Sundasciurus tenuis*, gray-bellied squirrel *Callosciurus caniceps* and rats *Rattus rattus*) based on trapping efforts. The purple, yellow, green and red points denote capture locations and gradient shading depicts areas of increased activity. The base camp is denoted by the green circle whereas the red starburst represents sightings of *Awang Besul*.

The current study shows that the frequency of CDV spillover to wildlife hosts and rare species has been increasing, with documentation of CDV infection in 22 families across five orders of taxa [24]. As molecular data from [9] suggests, the small mammal population in Kampung Besul Lama seeded the CDV infection to a Malayan tiger; the study uses heatmap illustration to enable visualization of the spatial distribution of different species and their activity based on live-trap capture coordinates in Kampung Besul Lama. Colored points were used to denote different species listed: Figure 4: Tree shrews (Scandentia), Figure 5: civets (Viverridae) and Figure 6: plantain squirrel *Callosciurus notatus*, slender squirrel *Sundasciurus tenuis*, gray-bellied squirrel *Callosciurus caniceps* and rats *Rattus rattus* (Rodents). Heatmap gradient denotes area of wildlife activity where colour of high intensity (whiter color) indicates higher wildlife abundance. Key features such as the base camp (green point) and confirmed sightings of the Malayan tiger *Awang Besul* (red starburst) are included to contextualize the spatial activity of wildlife species in relation to human activity and potential predator movement. The background satellite imagery provides landscape-scale context for habitat composition and fragmentation.

Figure 4 shows the heatmap of the areas of tree shrew *Tupaia glis* (Scandentia) activity around Kampung Besul Lama, particularly surrounding the forest edge and agricultural areas (orchards and oil palm plantations) providing attractive accessible food sources. Tree shrews are highly adaptable species, found in oil palm estates [25] and a diverse range of habitats and urban areas [26]. Based on the heatmap, we are able to see only minimal infiltration of the captured tree shrews’ foraging range into forests areas based on their daily movement of approximately 135 m [13]. However, the adaptability and abundance of tree shrews in diverse settings enables them to be an efficient reservoir for the CDV. Additionally, although tree shrew pairs are territorial [27], overlap in territories (male and female pair) [28] and multiple overlaps, where males’ territories overlap on multiple female territories [29], have also been reported, highlighting the potential for CDV to be maintained within this population. These factors highlight the potential role of tree shrews in sustaining CDV in an enzootic state and transmitting the virus to other susceptible species.

Figure 5 visualizes the spatial distribution and heatmap application of civets in Kampung Besul Lama. Civets have been reported to be susceptible to CDV in multiple instances in many different countries [30,31,32,33], making them an important component to consider for CDV transmission. Movement of civets between forested areas and cultivated areas in search of food [34] may contribute to a significant overlap in the domestic animal and wildlife interface. For instance, the spread of CDV to lions of the Serengeti was attributed to cross-species transmission from non-canid species, resulting in clinical disease in the lion population [24]. Civet heatmap concentration is higher around areas of human cultivation (lighter colored), increasing possibilities of CDV infection from domestic dogs. Thus, civets may be the main transmitter of CDV between domestic and wild species. Furthermore, civets have a large daily movement range and home range [35]. As seen from the heatmap, the captured civets’ projected roaming area includes almost 70% of the fragmented forest (East in Figure 5) where *Awang Besul* was suspected to originate. This could provide opportunity for viral transmission to rarer species only found in wild areas.

Figure 6 displays the heatmap analysis based on capture coordinates for order Rodentia sampled in this study, namely black rat *Rattus rattus*, plantain squirrel *Callosciurus notatus*, slender squirrel *Sundasciurus tenuis* and gray-bellied squirrel *Callosciurus caniceps*. Squirrels and rats occupy many habitats including urban, rural and forested areas [16,36,37]. Additionally, plantain squirrels have also been reported to be non-habitat-specific, thriving in gardens, cultivated vegetations and even towns [38]. CDV has been reported in order Rodentia [23]; thus, the versality of these species to thrive in different habitats including areas of human influence could contribute to the transmission of CDV from domestic species. In addition, squirrels normally coexist in the same ecological habitats, in which they reduce interspecies competition by occupying different vertical strata [37], contributing to interspecies CDV transmission. The proximity of the rodent heatmap to areas containing domestic animals would make the CDV infection to rodents possible. In addition, in this study, captured squirrels were overlapping in a habitat with the CDV-positive tree shrews [9], which suggests the possibility of disease transmission via fomites. Considering the factors above, efforts towards control and mitigation of CDV in small mammal populations must take into account the potentiality of CDV exposure and maintenance within the Rodentia order.

### 3.3. Potential Transmission Routes

**Figure 7:**
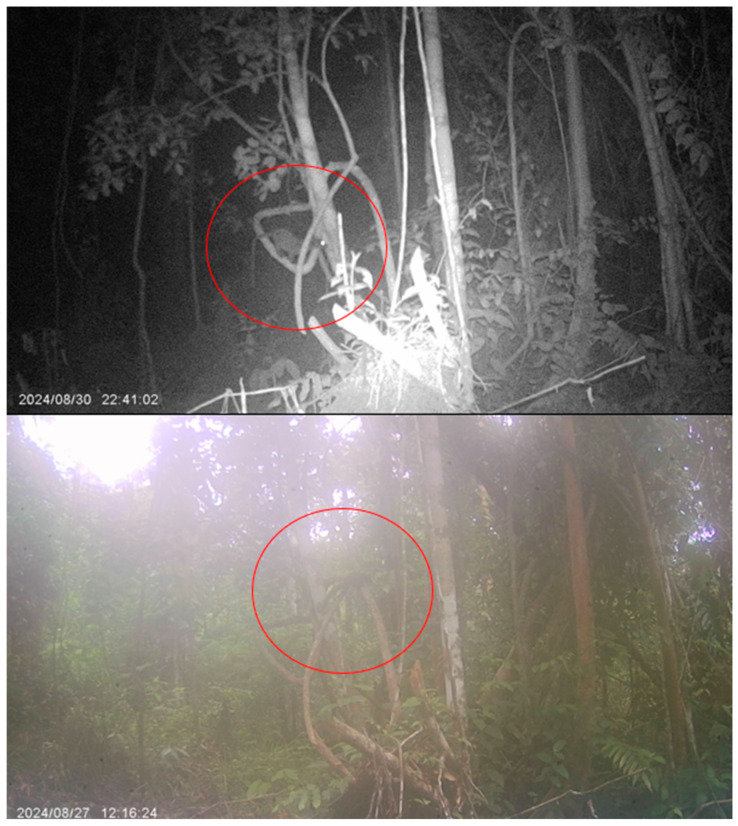
Tree shrew and civet utilizing the same pathway in Kampung Besul Lama, Terengganu.

Tree shrews have been discovered to be positive for the CDV within the proximity of Kampung Besul Lama [9], highlighting the role small mammals can play in the maintenance and transmission of wildlife disease. Additionally, hotspot areas of tree shrew activity around human settlements (Figure 4) may contribute to CDV spillover from domestic dogs to this species. However, tree shrews may not be the main transmitter of CDV towards *Awang Besul*. This is because of the small daily foraging range of tree shrews focused around areas of human settlements for food, with minimal infiltration into the surrounding forests. Although tree shrews were detected to be positive in Kampung Besul Lama, they may not be directly responsible for the disease transmission to *Awang Besul*, potentially transmitting the CDV to other small mammals in the vicinity such as civets. This is seen in Figure 7, where a tree shrew and a civet were documented in the same location (red circle), although two days apart, highlighting the potential for interspecies transmission between wildlife that share the same habitat.

**Figure 8:**
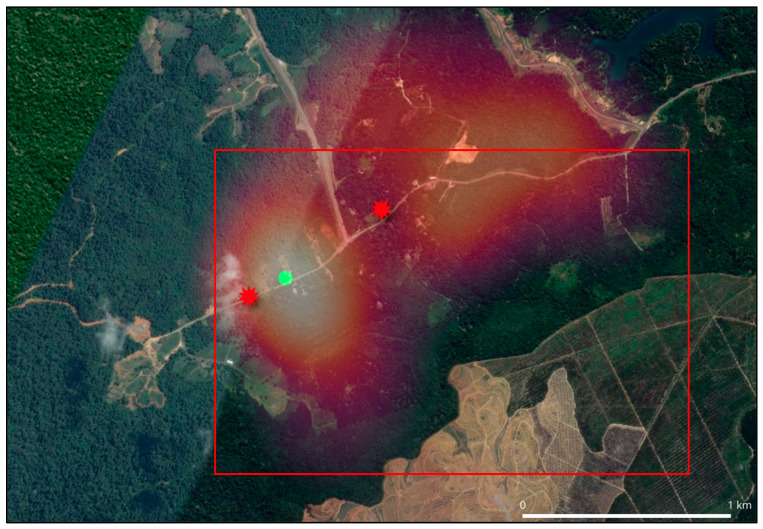
Deforestation on the habitat where locals say *Awang Besul* originated from based on previous sightings and roaring (red square). Image also depicts the sighting of the infected *Awang Besul* (red starburst) and our base camp (green circle).

Civets could be the main transmission route of CDV to *Awang Besul*. This is due to the movement of civets from forested areas towards areas of human cultivation in search of food, such as free-ranging chickens and fruits. Once infected, civet movement into forested areas could transmit the CDV to rare species such as the Malayan tiger either via predation or fomites. Furthermore, civets have the largest home range or daily movement range among all the sample species, highlighting their importance as potential disease spreaders and contributors of interspecies disease transmission. As seen in Figure 8, the civet heatmap shows civet movement into almost 80% of the forested area where *Awang Besul* initially resided (red square). Figure 8 also depicts the effects of indiscriminate logging and environmental degradation of prior tiger habitat, contributing to the movement of *Awang Besul* closer to areas of human settlement. Studies have suggested that modified habitats with reduced biodiversity, altered climates and fragmented forests may contribute to pathogen emergence in wildlife species [39]. Additionally, movement of *Awang Besul* could also have been influenced by the concurrent African swine fever outbreak in Malaysia, which severely impacted wild boar populations. Throughout the study, no wild boars were detected by the team and the villagers also reported a sudden lack of wild boars in the area. The reduction in a major food source for tigers could have led to the movement of *Awang Besul* closer to areas of anthropogenic influence in search of food, increasing exposure to canine distemper via the predation of free-roaming dogs or civets. This is supported by [40], who reported that the African swine fever outbreak in Malaysia has led to predator movements to areas of human settlements in search of food due to diminished prey availability.

### 3.4. Basic Reproduction Number

This study calculated the basic reproduction number (R_0_), which is defined as the average subsequent infections produced by an infected individual [18] for wildlife captured in Kampung Besul Lama. The population in Kampung Besul Lama was assumed to be equally susceptible to CDV, with a finite wildlife population of no deaths, births and migration. The estimated number of susceptible animals (n = 96) and area of Kampung Besul Lama (6.309 km^2^) were based on the number of species captured and the spatial distribution of all species using ellipsoidal calculations of the QGIS software. The transmission rate 0.1 [41] and recovery rate 0.1 [42] were utilized from the literature as it is extremely difficult to determine their precise values in wildlife which are scarce and open to bias [43]. Additionally, this is the first study attempting to research CDV in wildlife in Malaysia; thus, resources and data are scarce and subject to interpretation. The (R_0_) was calculated to be 15.21 in Kampung Besul Lama, which suggests an extremely high morbidity. Previous studies report that *Morbilliviruses* tend to have a higher (R_0_) of 12–18 [44], which supports the estimates. However, a high (R_0_) does not equate to high fatality, as it only describes potential transmission. The small mammal population may therefore act as competent reservoirs that amplify viral spread, even if clinical outcomes vary among species. Their close proximity to agricultural and forest edges further increases the likelihood of spillover events, sustaining transmission cycles within the ecosystem.

Additionally other factors could affect small mammal survivability, such as that CDV was already endemic within the local wildlife community. Thus, the outbreak that killed *Awang Besul* was a sporadic event that was not clinically significant to small mammal populations. Similar patterns were documented in the United States of America, where racoons were a reservoir for CDV and source of infections to zoo animals; however, the racoons displayed no clinical symptoms except for the occasional sporadic event [45]. If CDV had been endemic to Kampung Besul Lama or had circulated previously, the small mammal populations may have developed partial or population-level immunity, resulting in predominantly subclinical infections with minimal observable morbidity. This is further supported by observations in Kampung Besul Lama, where CDV-positive tree shrews exhibited no clinical symptoms [9], indicating the presence of subclinical disease within the community. Comparable events have also been documented in southern Africa where, despite high levels of CDV exposure, the wild dogs and lion populations were clinically unaffected [46], suggesting immunity or tolerance to the infection. Such patterns of subclinical disease are characteristic of wildlife hosts that have co-evolved with endemic pathogens, allowing them to sustain transmission without significant population-level mortality.

In addition, ref. [45] suggested that highly virulent strains of CDV would become extinct due to host mortality; hence, epizootics of less virulent strains with high morbidity and low mortality would occur. Kampung Besul Lama could have been subject to that event, leading to low mortality yet a high number of infections as supported by the (R_0_) estimates. The death of *Awang Besul* could be due to other physiological factors such as co-infection [47] or the variation in felid susceptibility to the CDV [3].

Additionally, ref. [9] only detected two positive samples of CDV in wildlife populations in Kampung Besul Lama, which may reflect a viral bottleneck. During CDV transmission, population bottlenecks can reduce viral genetic diversity, which is influenced by host immune pressures, restricted host availability or stochastic transmission events [48]. This causes the persistence and circulation of only a small number of viral lineages despite initial widespread transmission. This mechanism aligns with the high R_0_ (15.21) calculated in the area, suggesting an extensive earlier transmission with only a few surviving viral variants detected once the outbreak waned.

Furthermore, the low number of positive samples can be attributed to the typical bust and boom cycle of CDV infections [23]. The phase of high morbidity already occurred and concluded with the death of *Awang Besul*, in which the populations of small mammals were already recovering by the time the first CDV project in Malaysia was funded, which is typical of a sporadic event disease such as CDV. Additionally, the abundance of resource availability post-CDV event would have led to enhanced reproductive output among small mammals, positively affecting population recovery. A similar pattern of positive reproductive performance following a CDV event was documented in Danish foxes after a devastating CDV outbreak [49].

In conclusion, this study highlighted the role small mammals play in interspecies disease transmission. The heatmap for civets shows high infiltration into forested areas, making them a priority species to research for disease transmission. Additionally, the heatmap highlights the usage of edge habitats by multiple species and depicts overlap between small mammals and large predators within a human-modified landscape, contextualizing spatial associations across taxa. In addition, the study estimated the (R_0_) of Kampung Besul Lama, as well as put forth theories on the survivability of small mammal populations in the area. The spatial distribution range of small mammal species, especially civets in combination with the high (R_0_) of CDV estimated in Kampung Besul Lama, would shine light towards the infection and death of *Awang Besul*. However, further studies would need to be conducted utilizing GPS collaring across broader habitats to further understand the movement of civets to and from forested areas. Additionally, extensive laboratory experiments would need to be conducted to understand the physiology of unique small mammals in relation to CDV. Although ongoing theoretical research on R_0_ has extended to a range of complex modeling, including stochastic and finite systems, models with spatial structure or age structure and microparasite models [50], we note that the formula stated above is restricted to a very simple deterministic system for practical use in areas where thorough research has not been conducted. As CDV investigation in Malaysia is in its infancy, pioneer studies such as this will provide a useful baseline for future research and conservation strategies. Additionally, the results of this study can provide unique insight on the role of small mammals towards disease transmission, which can be critical for the control and mitigation of wildlife diseases. As small mammals susceptible to CDV are in abundance across different habitats, conservation and mitigation strategies would need to consider the role played by these species in disease transmission when formulating plans for tiger conservation.

## Figures and Tables

**Table 1:** Species captured and their capture coordinates in Kampung Besul Lama.

No	Sample ID	Species	Latitude	Longitude	Sex
1.	wDG01	Tree shrew *Tupaia glis*	4°42′38″ N	103°10′54″ E	M
2.	wDG02	Asian Palm Civet *Paradoxurus hermaphroditus*	4°42′32″ N	103°10′43″ E	M
3.	wDG03	Asian Palm Civet *Paradoxurus hermaphroditus*	4°42′33″ N	103°10′43″ E	M
4.	wDG04	Tree shrew *Tupaia glis*	4°42′35″ N	103°10′53″ E	M
5.	wDG05	Rat *Rattus rattus*	4°42′13″ N	103°10′34″ E	M
6.	wDG06	Asian Palm Civet *Paradoxurus hermaphroditus*	4°42′58″ N	103°11′09″ E	M
7.	wDG07	Asian Palm Civet *Paradoxurus hermaphroditus*	4°42′54″ N	103°11′00″ E	M
8.	wDG08	Rat *Rattus rattus*	4°42′14″ N	103°10′37″ E	M
9.	wDG09	Plantain squirrel *Callosciurus notatus*	4°42′33″ N	103°10′52″ E	M
10.	wDG10	Rat *Rattus rattus*	4°42′12″ N	103°10′39″ E	M
11.	wDG11	Plantain squirrel *Callosciurus notatus*	4°42′38″ N	103°10′54″ E	M
12.	wDG12	Plantain squirrel *Callosciurus notatus*	4°42′11″ N	103°10′46″ E	F
13.	wDG13	Asian Palm Civet *Paradoxurus hermaphroditus*	4°43′11″ N	103°11′03″ E	F
14.	wDG14	Gray-Bellied squirrel *Callosciurus caniceps*	4°42′33″ N	103°10′55″ E	M
15.	wDG15	Gray-Bellied squirrel *Callosciurus caniceps*	4°42′32″ N	103°10′50″ E	M
16.	wDG16	Gray-Bellied squirrel *Callosciurus caniceps*	4°42′26″ N	103°10′45″ E	F
17.	wDG17	Gray-Bellied squirrel *Callosciurus caniceps*	4°42′18″ N	103°10′50″ E	F
18.	wDG18	Gray-Bellied squirrel *Callosciurus caniceps*	4°42′15″ N	103°10′36″ E	F
19.	wDG19	Gray-Bellied squirrel *Callosciurus caniceps*	4°42′13″ N	103°10′43″ E	F
20.	wDG20	Gray-Bellied squirrel *Callosciurus caniceps*	4°42′37″ N	103°10′49″ E	M
21.	wDG21	Gray-Bellied squirrel *Callosciurus caniceps*	4°42′26″ N	103°10′40″ E	M
22.	wDG22	Rat *Rattus rattus*	4°42′10″ N	103°10′36″ E	M
23.	wDG23	Rat *Rattus rattus*	4°42′30″ N	103°10′45″ E	M
24.	wDG24	Slender squirrel *Sundasciurus tenuis*	4°42′18″ N	103°10′51″ E	M
25.	wDG25	Rat *Rattus rattus*	4°42′30″ N	103°10′45″ E	F
26.	wDG26	Rat *Rattus rattus*	4°42′31″ N	103°10′47″ E	M
27.	wDG27	Rat *Rattus rattus*	4°42′31″ N	103°10′47″ E	M
28.	wDG28	Rat *Rattus rattus*	4°42′32″ N	103°10′45″ E	F
29.	wDG29	Tree shrew *Tupaia glis*	4°42′24″ N	103°10′38″ E	F
30.	wDG30	Slender squirrel *Sundasciurus tenuis*	4°42′27″ N	103°10′45″ E	M
31.	wDG31	Slender squirrel *Sundasciurus tenuis*	4°42′33″ N	103°10′49″ E	M
32.	wDG32	Asian Palm Civet *Paradoxurus hermaphroditus*	4°43′12″ N	103°11′14″ E	M
33.	wDG33	Rat *Rattus rattus*	4°42′31″ N	103°10′45″ E	M
34.	wDG34	Rat *Rattus rattus*	4°42′32″ N	103°10′44″ E	F
35.	wDG35	Tree shrew *Tupaia glis*	4°42′12″ N	103°10′35″ E	M
36.	wDG36	Asian Palm Civet *Paradoxurus hermaphroditus*	4°42′27″ N	103°10′47″ E	M
37.	wDG37	Rat *Rattus rattus*	4°42′30″ N	103°10′47″ E	M
38.	wDG38	Tree shrew *Tupaia glis*	4°42′29″ N	103°10′41″ E	F
39.	wDG39	Plantain squirrel *Callosciurus notatus*	4°42′12″ N	103°10′37″ E	M
40.	wDG40	Plantain squirrel *Callosciurus notatus*	4°42′31″ N	103°10′48″ E	M
41.	wDG41	Rat *Rattus rattus*	4°42′32″ N	103°10′46″ E	M
42.	wDG42	Tree shrew *Tupaia glis*	4°42′29″ N	103°10′41″ E	M
43.	wDG43	Tree shrew *Tupaia glis*	4°42′23″ N	103°10′45″ E	F
44.	wDG44	Rat *Rattus rattus*	4°42′11″ N	103°10′35″ E	M
45.	wDG45	Asian Palm Civet *Paradoxurus hermaphroditus*	4°42′30″ N	103°10′57″ E	M
46.	wDG46	Slender squirrel *Sundasciurus tenuis*	4°42′33″ N	103°10′43″ E	M
47.	wDG47	Slender squirrel *Sundasciurus tenuis*	4°42′36″ N	103°10′49″ E	F
48.	wDG48	Slender squirrel *Sundasciurus tenuis*	4°42′15″ N	103°10′53″ E	M
49.	wDG49	Plantain squirrel *Callosciurus notatus*	4°42′27″ N	103°10′42″ E	M
50.	wDG50	Rat *Rattus rattus*	4°42′11″ N	103°10′44″ E	M
51.	wDG51	Rat *Rattus rattus*	4°42′10″ N	103°10′38″ E	M
52.	wDG52	Rat *Rattus rattus*	4°42′30″ N	103°10′44″ E	M
53.	wDG53	Plantain squirrel *Callosciurus notatus*	4°42′20″ N	103°10′39″ E	F
54.	wDG54	Tree shrew *Tupaia glis*	4°42′21″ N	103°10′42″ E	F
55.	wDG55	Rat *Rattus rattus*	4°42′33″ N	103°10′48″ E	M
56.	wDG56	Rat *Rattus rattus*	4°42′13″ N	103°10′36″ E	F
57.	wDG57	Rat *Rattus rattus*	4°42′30″ N	103°10′49″ E	F
58.	wDG58	Rat *Rattus rattus*	4°42′33″ N	103°10′44″ E	M
59.	wDG59	Tree shrew *Tupaia glis*	4°42′21″ N	103°10′42″ E	F
60.	wDG60	Tree shrew *Tupaia glis*	4°42′46″ N	103°10′53″ E	M
61.	wDG61	Slender squirrel *Sundasciurus tenuis*	4°42′24″ N	103°10′42″ E	M
62.	wDG62	Rat *Rattus rattus*	4°42′43″ N	103°10′54″ E	M
63.	wDG63	Rat *Rattus rattus*	4°42′45″ N	103°10′51″ E	F
64.	wDG64	Plantain squirrel *Callosciurus notatus*	4°42′19″ N	103°10′45″ E	M
65.	wDG65	Rat *Rattus rattus*	4°42′48″ N	103°10′50″ E	M
66.	wDG66	Tree shrew *Tupaia glis*	4°42′28″ N	103°10′48″ E	M
67.	wDG67	Rat *Rattus rattus*	4°42′42″ N	103°10′53″ E	F
68.	wDG68	Slender squirrel *Sundasciurus tenuis*	4°42′15″ N	103°10′38″ E	M
69.	wDG69	Plantain squirrel *Callosciurus notatus*	4°42′18″ N	103°10′50″ E	F
70.	wDG70	Long-tailed Giant Rat *Leopoldamys sabanus*	4°42′11″ N	103°10′44″ E	M
71.	wDG71	Tree shrew *Tupaia glis*	4°42′28″ N	103°10′48″ E	M
72.	wDG72	Rat *Rattus rattus*	4°42′43″ N	103°10′50″ E	F
73.	wDG73	Tree shrew *Tupaia glis*	4°42′36″ N	103°10′43″ E	F
74.	wDG74	Asian Palm Civet *Paradoxurus hermaphroditus*	4°42′47″ N	103°11′03″ E	M
75.	wDG75	Tree shrew *Tupaia glis*	4°42′28″ N	103°10′46″ E	F
76.	wDG76	Tree shrew *Tupaia glis*	4°42′32″ N	103°10′43″ E	M
77.	wDG77	Tree shrew *Tupaia glis*	4°42′12″ N	103°10′40″ E	M
78.	wDG78	Slender squirrel *Sundasciurus tenuis*	4°42′12″ N	103°10′35″ E	M
79.	wDG79	Rat *Rattus rattus*	4°42′45″ N	103°10′50″ E	M
80.	wDG80	Tree shrew *Tupaia glis*	4°42′11″ N	103°10′42″ E	M
81.	wDG81	Tree shrew *Tupaia glis*	4°42′12″ N	103°10′42″ E	F
82.	wDG82	Rat *Rattus rattus*	4°42′41″ N	103°10′51″ E	F
83.	wDG83	Rat *Rattus rattus*	4°42′48″ N	103°10′49″ E	F
84.	wDG84	Rat *Rattus rattus*	4°42′46″ N	103°10′56″ E	M
85.	wDG85	Rat *Rattus rattus*	4°42′49″ N	103°10′59″ E	F
86.	wDG86	Asian Palm Civet *Paradoxurus hermaphroditus*	4°42′17″ N	103°11′00″ E	M
87.	wDG87	Rat *Rattus rattus*	4°42′48″ N	103°11′02″ E	F
88.	wDG88	Slender squirrel *Sundasciurus tenuis*	4°42′31″ N	103°10′44″ E	F
89.	wDG89	Tree shrew *Tupaia glis*	4°42′15″ N	103°10′36″ E	F
90.	wDG90	Slender squirrel *Sundasciurus tenuis*	4°42′33″ N	103°10′45″ E	M
91.	wDG91	Malayan Civet *Viverra tangalunga*	4°42′47″ N	103°10′40″ E	M
92.	wDG92	Asian Palm Civet *Paradoxurus hermaphroditus*	4°43′05″ N	103°11′23″ E	M
93.	wDG93	Asian Palm Civet *Paradoxurus hermaphroditus*	4°43′08″ N	103°10′49″ E	F
94.	wDG94	Asian Palm Civet *Paradoxurus hermaphroditus*	4°43′00″ N	103°10′42″ E	M
95.	wDG95	Tree shrew *Tupaia glis*	4°42′13″ N	103°10′45″ E	F
96.	wDG96	Rat *Rattus rattus*	4°42′47″ N	103°11′02″ E	F

## Data Availability

The data presented in this study are available on request from the corresponding author.The locations and animals were available and included in this study. Some confidential data are restricted to protect endangered wildlife in the study area. The data can be available from the corresponding author for researchers who meet the criteria for access to confidential data.
